# 2-year results and myocardial impact of transapical mitral valve repair in patients with primary mitral regurgitation: an echocardiographic study

**DOI:** 10.1186/s13019-024-02827-3

**Published:** 2024-06-28

**Authors:** Chunqiang Hu, Zhenyi Ge, Wei Li, Wenzhi Pan, Zhengdan Ge, Weipeng Zhao, Dehong Kong, Daxin Zhou, Lai Wei, Xianhong Shu, Cuizhen Pan, Junbo Ge

**Affiliations:** 1 Department of Echocardiography, Shanghai Institute of Medical Imaging, Shanghai Institute of Cardiovascular Disease, Shanghai, China; 2 Department of Cardiology, Shanghai Institute of Cardiovascular Disease, Shanghai, China; 3 Department of Echocardiography, Zhongshan Hospital, Fudan University, 180 Fenglin Road, Shanghai, 200032 China

**Keywords:** Primary mitral regurgitation, Transcatheter edge-to-edge repair, ValveClamp, Echocardiography

## Abstract

**Background:**

There is limited data on the 2-year outcomes of transapical transcatheter edge-to-edge repair (TA-TEER) using the ValveClamp in patients with severe primary mitral regurgitation (MR) and its impact on myocardial deformation.

**Methods:**

From July 2018 to March 2021, 53 patients with symptomatic severe primary MR underwent TA-TEER were enrolled. The endpoint was the composite of all-cause mortality, recurrent 3 + or 4 + MR, or need for mitral surgery.

**Results:**

Among the 53 patients who had successfully ValveClamp implantation, 8(15.1%) reached the composite endpoint. Significant improvement in left ventricular (LV) end-diastolic volume, pulmonary artery systolic pressure, NYHA functional class, and MR severity were observed (*P* < 0.05 for all). Univariate Cox’s regression analysis revealed that LV end-diastolic volume index, LV end-systolic volume index, left atrial volume index, and pulmonary artery systolic pressure were associated with adverse events (*P* < 0.05 for all). On multivariate Cox regression analysis, left atrial volume index was independently associated with the endpoint (hazard ratio, 1.049; 95% CI, 1.009–1.091; *P* < 0.001) after adjustment for above echocardiographic parameters. LV global longitudinal strain and apical longitudinal strain in global and regional segments decreased at 30 days, but showed a recovery at 2 years with no significant difference compared to the baseline.

**Conclusion:**

TA-TEER using the ValveClamp presented favorable safety and efficacy at 2-year. Myocardial deformation impairment was observed at 30 days post-procedure, but did not persist at 2 years.

## Introduction

Mitral regurgitation (MR) is a common heart valvular disease, with a prevalence of 1.7% in the Western population, accompanied by considerable morbidity and mortality [1]. Moderate or severe MR affects an even higher proportion, approximately 13.3%, of individuals older than 75 years [1–4]. Symptomatic severe primary MR (PMR) is the most common indication for isolated mitral valve surgery in Western countries [5]. However, patients with severe PMR, due to advanced age or high surgical risk, are often denied surgery. Transcatheter edge-to-edge mitral valve repair (TEER) has emerged as a reasonable therapeutic option for these patients [6–8]. Percutaneous femoral venous transseptal access is believed to be the least invasive and thus is frequently chosen as the preferred approach. Nevertheless, it is noteworthy that the transapical approach using the ValveClamp device has been previously reported in a small-scale and multi-center research with comparable procedural success, safety, and short-term efficacy [9, 10]. Transapical TEER is easy-operate to achieve the coaxiality to the mitral valve under the guidance of real-time three-dimensional transesophageal echocardiography due to short and straight route of transapical approach and the large capture range of the ValveClamp. However, the transapical approach leads to a localized injury at the apex. Echocardiographic strain analysis is the commonly applied imaging modality for identifying subclinical myocardial motion impairment. The aim of this study was to report the mid-term (2-year) outcomes in a consecutive series of patients treated by transapical TEER (TA-TEER) and to investigate whether transapical approach impairs myocardial deformation utilizing echocardiographic analysis.

## Method

The primary endpoint was defined as the composite of all-cause death, recurrent MR grade ≥ 3+, or necessity for mitral surgery. In this prospective observational study, a total of 53 consecutive patients with moderate-to-severe or greater PMR, including 20 patients who were involved in the first-in-human study at our center, were enrolled between July 2018 and March 2021 at Zhongshan Hospital, Fudan University. These patients underwent TA-TEER using the ValveClamp system developed by Hanyu Medical Technology, Shanghai, China. The inclusion and exclusion criteria have been previously reported [10]. The specified inclusion criteria were as follows: (1) symptomatic (New York Heart Association [NYHA] functional class ≥ II) moderate-to-severe or greater PMR, (2) the etiology of MR is prolapse or flail; (3) primary jets originating from A2 or P2 mitral scallops, and (4) high-risk surgical individuals evaluated by the heart team, as defined by the Society of Thoracic Surgeons score (STS) > 8%, or by the presence of more than 2 concomitant major organ system compromise not to be improved post-procudurally. Patients who presented with any of the following conditions were excluded: (1) rheumatic disease or mitral leaflet perforation, (2) mitral leaflet calcification or previous mitral intervention, (3) significant concomitant valvular disease, and (4) life expectancy < 12 months. This study was approved by the Institutional Review Board of Zhongshan Hospital, Fudan University (2019-002R) and conducted in accordance with the Declaration of Helsinki. Informed consent was obtained from all study participants. Their subsequent information was anonymized and deidentified.

Transthoracic echocardiography was performed upon admission, prior to discharge from the hospital, and at the 30-day, 6-month, 12-month, and 2-year follow-up intervals. Pre-TA-TEER, 30-day and 2-year echocardiograms were retrospectively analyzed by a core laboratory at Zhongshan Hospital, Fudan University. Biplane Simpson’s method was employed to measure left ventricular (LV) end-diastolic volume (LVEDV), LV end-systolic volume (LVESV), LV ejection fraction (LVEF), and left atrial volume (LAV). LVEDV index (LVEDVI), LVESV index (LVESVI) and LAV index (LAVI) were derived by dividing the LVEDV, LVESV and LAV by individual’s body surface area (BSA) respectively. Vena contracta width (VCW) was determined by averaging measurements obtained from apical four-chamber and apical two-chamber views. Mitral valve area (MVA) was measured using multiplanar reconstruction on three-dimensional volumes of the mitral valve. Mean mitral gradient (MVG) were measured using continuous-wave Doppler from the apical four-chamber view. LV longitudinal strain, encompassing LV global longitudinal strain (LV GLS), as well as global and segmental apical longitudinal strain, was evaluated using Qlab 13.0 (Philips Healthcare, Andover, MA, USA) with dedicated algorithms [11]. The LV longitudinal strain measurements were denoted as absolute percentage values to simplify the interpretation of myocardial deformation. The severity of MR was evaluated utilizing the integrated multi-parameter algorithms in accordance with recommended guidelines [12]. 

The structural details of the ValveClamp system and procedural steps of TA-TEER had been described previously [9, 10, 13]. The ValveClamp system comprises front and rear clamps with arm lengths of 9 mm and 10 mm, respectively, and a closed ring. Its design aims to expand leaflet capture range, surpassing that of the MitraClip despite similar arm dimensions. Transapical implantation involves the several steps: (1) Determine the appropriate apical puncture site using transthoracic echocardiography, (2) A 16 F introducer sheath was inserted into the left atrium guided by standard bi-commissural two-chamber and orthogonal long-axis views on transesophageal echocardiography. The clamp was then delivered through the introducer sheath into the left atrium and opened. (3) After ensuring that the clamp arms are positioned towards the center of the regurgitant jet and perpendicular to the mitral coaptation line, gently retrieve back the rear clamp into the LV to hold the leaflets. (4) Pull back the front clamp to grasp the leaflets, followed by advancing the closed ring to approximate and reinforce the clamp. Before releasing, the insertion length of leaflets, severity of residual MR, and MVG were evaluated. Procedural success was defined as successful access, delivery, and correct clamping. All patients were followed up via telephone call or outpatient visits at 30 days, 6 months, 1 year, and 2 years post-discharge.

Categorical variables were presented as frequency (percentages) and assessed for differences between baseline and follow-up using χ^2^ test. Continuous variables were presented as median (25th–75th IQ) and compared between baseline and follow-up using Wilcoxon matched-pairs signed rank test. Univariate analyses were conducted using the Cox proportional hazards model to calculate hazard ratio (HR) of prespecified characteristics, after which variables with P value < 0.05 were entered into a multivariable model. A two-tailed P value less than 0.05 was regarded as statistically significant. All statistical analyses were performed using SPSS 25 (IBM Crop, Armonk, NY, USA).

## Results

Baseline clinical and echocardiographic characteristics of the study population are presented in Table 1. The study recruited 53 participants (aged 75(70, 80) years, 41.5% male) who had moderate-to-severe or severe PMR, with median Society of Thoracic Surgeons score (STS) of 6.74% (6.19%, 7.87%). The comorbidities were as follows: 15(28.3%) patients had atrial fibrillation, 20(37.7%) had coronary artery disease, 25(47.2%) had hypertension, 7(13.2%) had chronic obstructive pulmonary disease, and 6(11.3%) had a history of stroke or transient ischemic attack. All patients manifested symptoms, with 35(66.0%) classified as NYHA functional class III, and 11(20.7%) classified as class IV.

**Table 1:** Baseline and echocardiographic characteristics

Parameters	*n* = 53
**Baseline**	
Age, years	75(70, 80)
Sex, male	22(41.5%)
BSA, m^2^	1.56(1.43, 1.72)
NYHA functional class	
III	35(66.0%)
IV	11(20.7%)
STS, %	6.74(6.19, 7.87)
AF	15(28.3%)
Hypertension	25(47.2%)
CAD	20(37.7%)
COPD	7(13.2%)
DM	6(11.3%)
Stroke/TIA	6(11.3%)
Nt-proBNP	769.0(356.3, 1774.0)
TnT	0.15(0.06, 0.28)
Creatinine	90.5(69.0, 109.0)
eGFR	60.5(45.8, 83.0)
TG	1.23(0.83, 1.72)
**Echocardiography**	
LVEDD, mm	52.5(49.0, 55.0)
LVESD, mm	32.0(30.0, 34.8)
LVEDVI, mL/m^2^	56.6(51.8, 71.8)
LVESVI, mL/m^2^	19.3(16.7, 23.6)
LVEF, %	66.0(63.3, 70.0)
LV GLS, %	24.3(21.4, 26.4)
LAVI, mL/m^2^	44.6(35.0, 58.3)
MVG, mmHg	3.0(2.0, 4.4)
MVA, cm^2^	3.77(3.61, 4.03)
VCW, mm	8.6(8.0, 9.6)
MR severity	
3+	1(1.9%)
4+	52(98.1%)
TR ≥ 3+	7(13.2%)
sPAP, mmHg	50.0(41.0, 68.0)

BSA = body surface area; NYHA = New York Heart Association; AF = atrial fibrillation; STS: Society for Thoracic Surgeons risk score; CAD = coronary artery disease; COPD = chronic obstructive pulmonary disease; DM = diabetes mellitus; TIA = transient ischemic attack; NT-proBNP = N-terminal brain natriuretic peptide; TnT = cardiac troponin; eGFR = estimated glomerular filtration rate; TG = triglyceride; LVEDD = left ventricular end-diastolic diameter; LVESD = left ventricular end-systolic diameter; LVEDVI = left ventricular end-diastolic volume index; LVESVI = left ventricular end-systolic volume index; LVEF = left ventricular ejection fraction; GLS = global longitudinal strain; LAVI = left atrial volume index; MVG = mean mitral valve gradient; MVA = mitral valve area; VCW = vena contracta width; MR = mitral regurgitation; TR = tricuspid regurgitation; sPAP = pulmonary artery systolic pressure

### Procedure

The TA-TEER procedure was performed on all 53 patients, with a median catheterization duration of 43.5(39.0, 54.8) minutes. Unfortunately, one patient required a transition to surgical repair as it was not possible to reduce the severity of MR to a favorable extent. Out of these 53 successful cases, 48 patients underwent the procedure employing a single clamp, whereas the remaining 5 patients underwent the implantation of two clamps. No deaths were recorded throughout the perioperative duration.

### Echocardiographic measurements

**Fig. 1:**
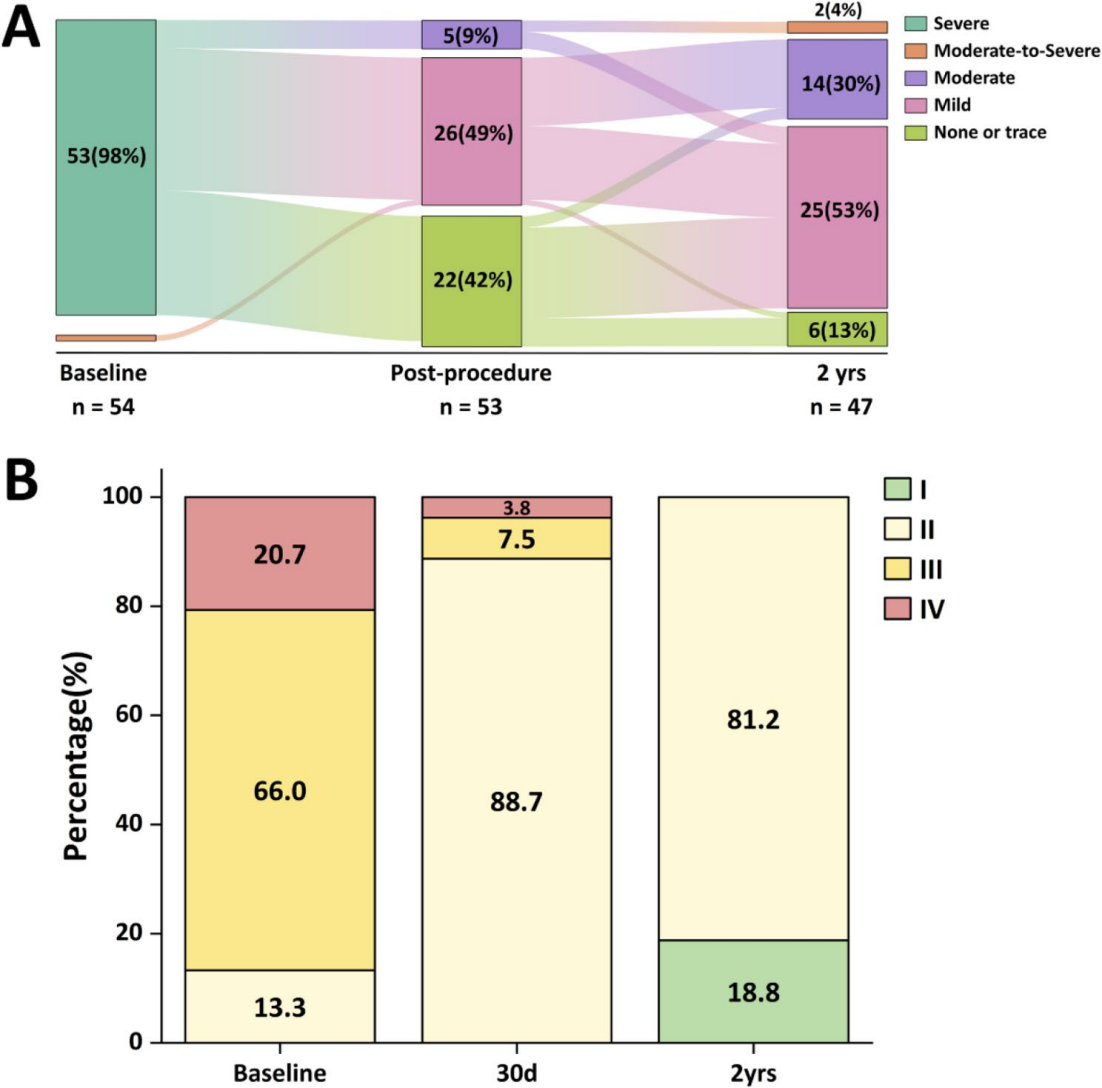
The severity of mitral regurgitation and NYHA functional class at baseline and follow-up

Transthoracic echocardiographic images were available for the comprehensive evaluation of MR severity, dimensions of the cardiac chambers, and LV function at baseline, post-procedure, 30-day and 2-year follow-up. Although all of patients exhibited MR grade of moderate-to-severe or severe at baseline, it was found that 22 (42%) patients had none or trace MR, 26(49%) had mild MR, and 5(9%) had moderate MR immediately after the procedure. At 2-year follow-up, 6(13%) patients had none or trace MR, 25(53%) had mild MR, 14(30%) had moderate MR, 2(4%) had moderate to severe MR (Fig. 1A).

**Fig. 2:**
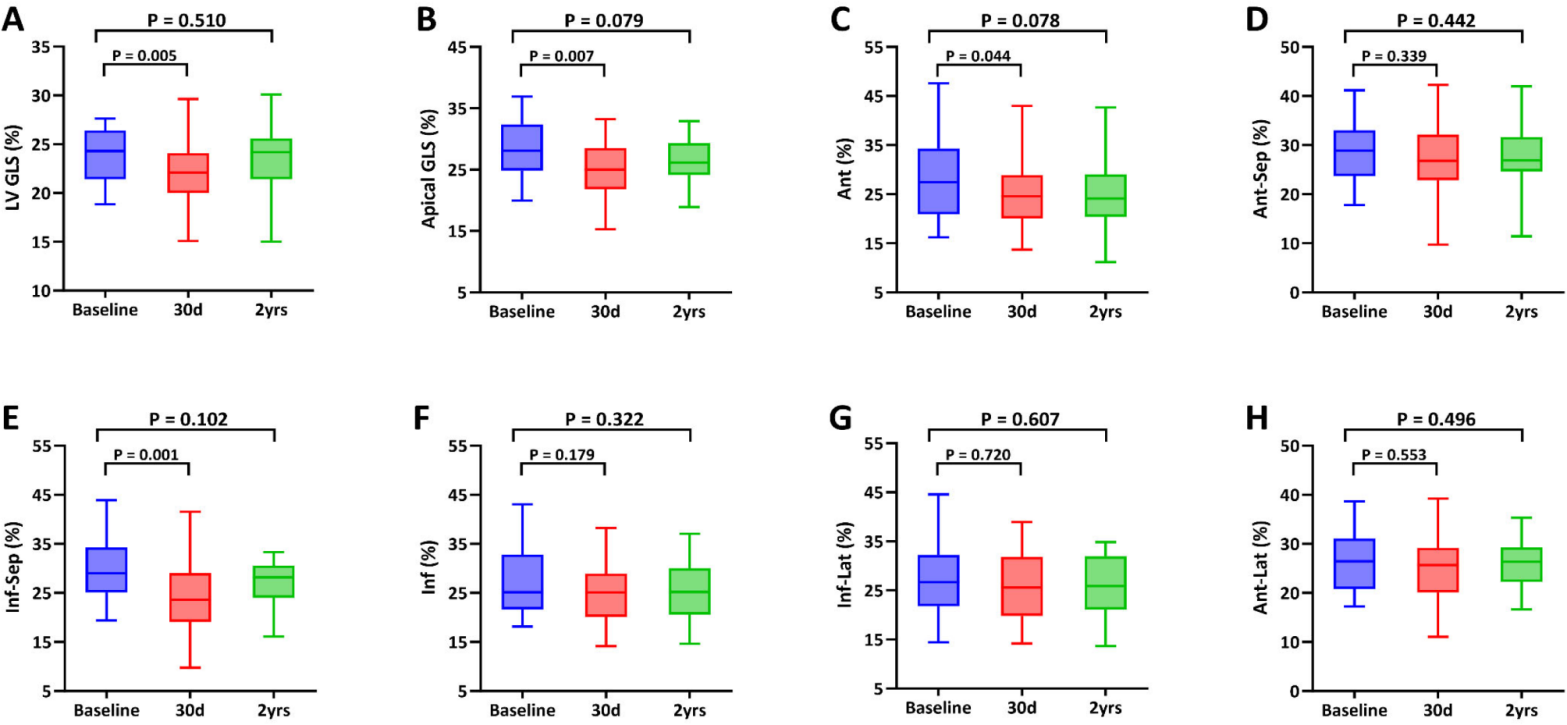
Left ventricular global longitudinal strain (LV GLS) and apical longitudinal strain at baseline, 30d, and 2-year follow-up. (**A**) LV GLS; (**B**) LV apical GLS; Apical longitudinal strain was performed in all segments, including anterior (**C**), anterior septal (**D**), inferior septal (**E**), inferior (**F**), inferior lateral (**G**), and anterior lateral segment (**H**). GLS = global longitudinal strain; Ant = anterior; Ant-Sep = anterior septal; Inf-Sep = inferior septal; Inf = inferior; Inf-Lat = inferior lateral; Ant-Lat = anterior lateral

For echocardiographic variables, a significant decrease in LVEDVI, LVESVI, and pulmonary artery systolic pressure (sPAP) was observed at 2-year follow-up (*P* < 0.05 for all) (Table 2). In contrast, MVG at 2 years was 4.0(3.0, 5.1) mmHg compared with 3.0(2.0, 4.4) mmHg at baseline (*P* = 0.006). Although a slight decline was observed in LVEF at 30 days [65.8%(63.0%, 70.0%) vs. 64.2%(59.2%, 68.2%), *P* = 0.001], there was no significant change over the course of 2-year follow-up [65.8%(63.0%, 70.0%) vs. 65.1%(61.9%, 68.7%), *P* = 0.263]. Myocardial deformation imaging revealed that LV GLS had decreased at 30 days [ 24.3% (21.4%, 26.4%) vs. 22.1% (20.0%, 24.1%), *P* = 0.003], while showed no statistically difference at 2 years [24.3% (21.4%, 26.4%) vs. 24.2% (21.4%, 25.6%), *P* = 0.393]. Furthermore, among the 45 patients who were free from endpoint events, 40 had normal LV GLS at 2 years, while 49 out of 53 patients had normal LV GLS at baseline, indicating no differences (*P* = 0.728). LV apical global longitudinal strain, as well as the apical longitudinal strain of anterior and inferior-septal segment, decreased at 30-day post-procedurally (*P* < 0.05 for all), but recovered to baseline level at 2-year follow-up (*P* > 0.05 for all). During the 2-year study period, no notable difference in longitudinal strain of other apical segments was observed when compared to the preoperative measurements, respectively (Fig. 2).

**Table 2:** Comparison of echocardiographic parameters at baseline and 2y

	Baseline	30d	2yrs	*P* value^a^	*P* value^b^
LVEDVI, mL	56.3(51.4, 69.8)	47.0(39.3, 54.9)	51.2(41.6, 59.4)	**< 0.001**	**0.002**
LVESVI, mL	19.3(16.2, 23.3)	17.7(13.5, 20.8)	18.2(14.5, 20.1)	**0.003**	**0.047**
LVEF, %	65.8(63.0, 70.0)	64.2(59.4, 68.2)	65.1(61.9, 68.7)	**0.001**	0.263
LV GLS, %	24.3(21.4, 26.4)	22.1(20.0, 24.1)	24.2(21.4, 25.6)	**0.003**	0.393
MVG, mmHg	3.0(2.0, 4.4)	3.0(3.0,4.2)	4.0(3.0, 5.3)	0.191	**0.006**
sPAP, mmHg	50.0(41.0, 68.0)	36.0(31.0,41.5)	39.0(33.0, 46.0)	**< 0.001**	**< 0.001**

Bold values indicate statistical significance

^a^Comparison between baseline and 30 days, ^b^Comparison between baseline and 2 years

LVEDVI = left ventricular end-diastolic volume index; LVESVI = left ventricular end-systolic volume index; LVEF = left ventricular ejection fraction; LV GLS = left ventricular global longitudinal strain; MVG = mean mitral valve gradient; sPAP = pulmonary artery systolic pressure

### Clinical outcome

There were considerable improvements in NYHA functional class at the 30-day time period, and remained evident at the end of the 2-year follow-up period. At baseline, 46 patients (86.7%) were classified in NYHA class III/IV, which decreased to 11.3% after 30 days and ultimately eliminated after 2 years, *P* < 0.001) (Fig. 1B). Nevertheless, 8 patients (18.9%) reached the predetermined endpoint, and 4 patients died resulting in an all-cause mortality of 7.5%. During the 2-year study period, 1 patient died before hospital discharge, while another died 14 days after the implantation of the clamp. Furthermore, two additional patients died within the 1-year and 2-year follow-up periods. 2 patients were referred for surgical mitral valve repair due to recurrent 4 + MR in one case, and device malposition 30 days after the procedure in the other case. Additionally, the other 2 patients experienced recurrent 3 + MR.

At univariable Cox regression analysis, several echocardiographic variables were found to be significantly associated with the predetermined clinical outcomes. Specifically, greater sPAP [HR, 1.057; 95% confident interval (CI), 1.013–1.103; *P* = 0.011], LAVI (HR, 1.059; 95% CI, 1.019–1.101; *P* = 0.003), LVEDVI (HR, 1.088; 95% CI, 1.024–1.157; *P* = 0.007), LVESVI (HR, 1.131; 95% CI, 1.008–1.269); *P* = 0.036) were identified as predictors of the composite endpoints in this study (Table 3). Besides, those individuals who were subsequently enrolled following the initial first-in-human study experienced a reduction in endpoint events (HR, 0.159; 95% CI, 0.028–0.910; *P* = 0.039). On multivariate Cox regression analysis considered the above echocardiographic parameters, LAVI was predictive of the endpoints (HR, 1.049; 95% CI, 1.009–1.091; *P* < 0.001).

**Table 3:** Univariate cox regression analysis for identifying adverse events

Variable	HR	95% CI		*P* Value
		Lower	Upper	
Age	1.117	0.974	1.281	0.113
Sex, male	0.918	0.205	4.104	0.911
Heart Rate	0.997	0.956	1.04	0.892
Procedure Duration	1.023	0.990	1.058	0.179
STS	0.986	0.743	1.309	0.925
LVEDD	1.022	0.881	1.184	0.777
LVESD	1.027	0.893	1.179	0.712
LVEDVI	1.088	1.024	1.157	**0.007**
LVESVI	1.131	1.008	1.269	**0.036**
LVEF	1.139	0.935	1.387	0.197
LAVI	1.059	1.019	1.101	**0.003**
VCW	1.158	0.763	1.759	0.490
MVA	2.358	0.856	6.495	0.097
MVG	1.132	0.805	1.593	0.475
sPAP	1.057	1.013	1.103	**0.011**
LV GLS	0.977	0.744	1.282	0.865
FIH (yes/no)	0.159	0.028	0.910	**0.039**

LVEDD = left ventricular end-diastolic diameter; LVESD = left ventricular end-systolic diameter; LVEDVI = left ventricular end-diastolic volume index; LVESVI = left ventricular end-systolic volume index; LVEF = left ventricular ejection fraction; LAVI = left atrial volume index; VCW = vena contracta width; MVA = mitral valve area; MVG = mean mitral valve gradient; sPAP = pulmonary artery systolic pressure; LV GLS = left ventricular global longitudinal strain; FIH = first-in-human

## Discussion

In this prospective single-arm study that included all consecutive patients undergoing TA-TEER for severe PMR, the primary findings were as follows: (1) the safety profile of the ValveClamp system was favorable, despite the advanced age and existence of significant comorbidities in patients that would typically make them less suitable candidates for open heart surgery; (2) this study demonstrated encouraging clinical outcomes following a span of 2 years, characterized by a reduction in MR severity, and both improvement in cardiac function and quality of life; (3) the temporary impairment in myocardial deformation was not sustained after a period of 2 years.

There is an increasing utilization of TEER in patients with intricate anatomy of mitral valve, along with a reinvigorated inclination to expand its application to individuals with lower surgical risk [14, 15]. In order to promote the exploit of TEER therapy to more challenging conditions, it is imperative to substantiate their clinical efficacy, either by validating the comparability to or superiority over the conventional open surgery, for eligible patients suffering from MR.

Several cardiac interventions have explored transapical access, and transapical transcatheter aortic valve replacement (TA-TAVR) is most often used when peripheral venous access is poor. Transapical mitral neochordae implantation with the NeoChord DS1000 (NeoChord Inc, St. Louis Park, MN, USA) device showed feasibility and good outcomes up to 5 years in patients with severe PMR [16, 17]. It may serve as a valid alternative to conventional surgery in selected patients with high likelihood of success based on clinical and anatomical factors. The ValveClamp device is an innovative TEER system via apical access engineered to optimize procedure process with a median catheterization duration of 43.5(39.0, 54.8) mins in this study, implying that the requisite learning curve for doctors to proficiently deploy this device is comparatively short. Early positive findings regarding the safety and efficacy of the ValveClamp in treating patients with severe PMR have been reported [18]. Similarly, the safety outcomes in this study were comparable despite the advanced age and significant comorbidities among enrolled patients. In this cohort aged 75(70, 80) years, we did not observe cases of periprocedural deaths, procedure-related stroke, life-threatening bleeding, or reintervention. Moreover, despite the median STS was 6.74%, we observed a 30-day mortality rate of 3.7%. A recent real-world interventional series with the MitraClip device reported an in-hospital death rate of 1.1% and a 30-day death rate of 2.7% which is comparable to our results [8]. This data suggests the potential safety of the TA-TEER using the ValveClamp device.

Furthermore, the consistent elimination of MR in this study was accompanied with LV reverse remodeling, demonstrated by a decrease in LVEDVI. The preservation of LVEF and LV GLS, along with a reduction in sPAP, were seen in consistence with an improvement in symptoms, as indicated by the NYHA functional class. These findings provided compelling evidence to substantiate the efficacy of the ValveClamp device. However, the MVG was worse at 2 years compared to baseline in our study. Similar to other TEER device, the ValveClamp would narrow MVA, subsequently resulting in a higher MVG. The median of MVG [4.0 (3.0, 5.1) mmHg] is relatively low gradient which had the better clinical outcomes compare elevated gradient (> 5 mmHg) [19]. 

It has been reported that the development of apical pseudoaneurysm after TA-TAVR [20, 21]. Additionally, myocardial strain based on cardiac magnetic resonance revealed a significant abnormality in apical LV function characterized by a decrease in apical peak systolic longitudinal strain and peak systolic radial strain at 3-month follow-up in all TA-TAVR patients [22]. An echocardiographic study revealed that 28% of the patients experienced deterioration in myocardial function following the procedure, and half of these patients demonstrated a recovery of apical function while exhibiting a lower long-term follow-up LVEF (50% vs. 60%, *P* = 0.045) [23]. Although there was some extent of apical myocardial injury reported after TA-TAVR, no impairment was observed in the apical global longitudinal strain or apical segmental strain after TA-TEER. In our study, a 16 F delivery sheath was used to introduce the clamp device to left atria, which was smaller compared to the 22–24 F sheath used in the TA-TAVR procedure. Therefore, patients who underwent TA-TEER may experience fewer injuries during the post-procedure period and follow-up. Furthermore, the absence of pacing and a shorter procedure time may collectively reduce the risk of injury to the apical myocardium.

There is evidence indicating that LAVI was associated with clinical outcomes across a wide of cardiovascular diseases, such as hypertrophic myocardiopathy, secondary MR, and acute myocardial infarction [24–29]. Specifically, LA enlargement exhibited a strong correlation with the severity of MR, whereas LA reverse remodeling is a goal of surgical repair. Given the established role of LAVI, LAVI was also associated with the composite endpoint outcomes after TA-TEER with a HR of 1.049 (95% CI: 1.009–1.091), providing clinicians with valuable information to guide patient management and decision-making. This study sheds light on the prognostic role of the aforementioned echocardiographic variables, offering novel evidence to identify suitable candidates for achieving optimal outcomes. In our previous study, patients with PMR with a tenting volume index ≥ 0.82 mL/m^2^ are more likely to have acute residual MR 2 + and recurrent moderate to severe or greater MR [10]. Additionally, patients with severe peripheral venous disease may opt for TA-TEER instead. Further investigation into other specific criteria is warranted. The early study of TA-TEER demonstrated a 100% of device success rate and all patients were free from adverse events (death, surgery for valve dysfunction, or recurrent MR 3+) at 30 days, with one patient experiencing recurrent MR 3 + at 1 year [18]. Our study indicated that there was a favorable improvement in clinical outcomes among severe PMR patients who undergone TA-TEER after the first-in-human study [18]. This improvement can be attributed, in part, to the increased acknowledge of the ValveClamp device and procedural process by interventional cardiologists and the improved navigational skills by intra-procedure echocardiographers. Therefore, it is hypothesized that the TA-TEER procedure using the ValveClamp device possesses a relatively short learning curve, and mastery of the procedure and navigation skills may significantly improve the procedural outcomes.

## Limitations

Several limitations of our study were noted as follows: (1) The current study provides the 2-year follow-up outcome data of the patient cohort treated with TA-TEER. However, it is important to acknowledge that this study is a relatively small, single-arm interventional study. There were no control groups for comparison of our findings, such as surgically or medically treated groups; (2) The study exclusively enrolled individuals with lesions involving A2/P2 scallops, therefore, the safety and efficacy of the ValveClamp in patients with involvement of the commissural regions has yet to be elucidated; (3) There were no cardiovascular magnetic resonance imaging (CMR) examination to better characterize the injury and fibrosis of myocardia pre- and post-procedure. (4) Due to the limited number of patients who reached the endpoint, the result of multivariate Cox regression analysis may not be robust.

## Conclusions

TA-TEER using the ValveClamp in patients with PMR demonstrated a sustained reduction in MR and associated improvement in cardiac function at 2 years. Furthermore, no significant myocardial injury was noted due to the transapical access at 2-year follow-up.

## Data Availability

No datasets were generated or analysed during the current study.

## Abbreviations

MR: Mitral regurgitation
PMR: Primary MR
TEER: Transcatheter edge-to-edge mitral valve repair
TA-TEER: Transapical TEER
NYHA: New York Heart Association
LV: Left ventricular
LVEDV: LV end-diastolic volume
LVESV: LV end-systolic volume
LVEDVI: LV end-diastolic volume index
LVESVI: LV end-systolic volume index
LVEF: LV ejection fraction
LAV: Left atrial volume
LAVI: LAV index
VCW: Vena contracta width
MVA: Mitral valve area
MVG: Mean mitral valve gradient
LV GLS: LV global longitudinal strain
HR: Hazard ratio
sPAP: Pulmonary artery systolic pressure
CMR: Cardiovascular magnetic resonance imaging
